# Local Anæsthesia by Chloroform—Failure—Disappearance of the Patient

**Published:** 1854-10

**Authors:** 


*Local Anæsthesia by Chloroform—Failure—Disappearance of the Patient.*—Having read the accounts in the Peninsular and other journals of the power of chloroform topically applied to produce local insensibility, I determined to embrace the first opportunity to try it, and if successful, as I doubted not I should be, I was resolved to devise means of using it thus in every case where it was possible, and thus diminish the risk of having a patient drop from my hands into the grave, in the sudden and appalling manner which sometimes occurs. The first case which I deemed a proper one, came under my notice last December. The patient came into my office limping, and on withdrawing his boot, showed me one of those common cases where the nail of the great toe grows into the flesh so as to produce a very painful swelling, rendering walking and wearing of the boot intolerable. After examining it, I recommended the operation which has lately come into use for such cases, viz., slicing off the projecting mass of flesh from the side where the nail was imbedded. The patient inquired if it would hurt? I informed him that it would. He then asked advice about taking chloroform. I told him that for so slight an operation it was not advisable to run the risk of taking it internally; but that I would apply it externally, so as to destroy the sensibility of the toe alone, and thus save him from both pain and danger. I then proceeded, according to the directions which I had seen published, to produce the local insensibility. I dipped some lint in chloroform and wrapped it round the toe, and then enclosed the whole in oiled silk to confine the vapor. The results were ludicrous. In the first place, I found that oiled silk will no more confine the vapor of chloroform than a sieve will confine water, or flax fire. It crumbled to pieces almost the instant the vapor touched it. Failing in this, I took a wide-mouthed vial, and thrust the toe with its envelope, into it, and thus succeeded in securing a close atmosphere of the fluid around it.

I now waited with great confidence for the wished-for insensibility to pain, but was doomed again to disappointment. The surface exposed to the vapor soon began to smart and burn most intolerably. I exhorted the patient to bear it coolly, assuring him it was a sign that the agent was taking effect; that the smarting would soon subside, and be succeeded by a pleasant quietness. Thus encouraged, he bore it manfully for some time, but still it increased to such an extent that at length human endurance could bear it no longer; and in spite of my remonstrances, he tore off the coverings to get relief. I was astonished at such a result, after the accounts I had read, and still thought that it must be owing to the tender and irritated state of the skin, and that I should certainly produce insensibility if I could induce the patient to submit a little longer. With this view, I deadened the sensibility of the surface by rubbing it over with nitrate of silver, and then re-applied the chloroform. This at first worked admirably. The vapor no longer produced pain, and the patient's countenance lighted up with joyful expectation. Soon, however, the smarting began to return, but rather slowly, and I made up my mind that I should now succeed; but as time was passing away, and I had other patients to visit, I told my present one to remain quiet while I was gone; gave him the chloroform bottle with directions to add a few drops every ten minutes to the dressing, and that I would return in an hour, by which time the insensibility would be complete, and I would proceed to operate.

After about an hour, I returned to my office. There sat the patient writhing with anguish in his chair, still resolutely holding his toe in the vial and adding new chloroform every ten minutes according to directions. I asked him if he felt easy yet; he informed me in very explicit language that he did not, and that he had as lief hold his toe in the fire. As the application had now been continued two hours, I gave it up, half provoked and half laughing at my egregious failure. I took off the dressings, and satisfied myself, by examination, that the skin retained all its sensibility to external touch. I informed the patient that I had not succeeded in producing insensibility, a fact of which he was already well aware; whereupon he drew on his boot and departed, since which I have not seen him.

Whether my failure was owing to my ignorance of the proper cases adapted to such treatment; or of the proper mode of application—or whether the local anæsthetic power of chloroform is a delusion, I am not able to say. If the experience of any physician who reads this has been different from mine, I hope he will communicate it to the world; for if the local anæsthesia can really be obtained, it is a discovery of the utmost value, and should be made public, with such details as will enable all practitioners to avail themselves of it.—*Boston Med. and Surg. Jour. from Peninsular Jour. of Med.*

